# Errors in Figures and Supplement

**DOI:** 10.1001/jamanetworkopen.2024.29990

**Published:** 2024-07-31

In the Original Investigation titled “Biomarker Testing in Patients With Unresectable Advanced or Recurrent Non–Small Cell Lung Cancer,”^1^ published December 15, 2023, the first 2 labels in the x-axis of Figure 1 were inadvertently transposed; the bars indicating multigene testing were inadvertently labeled as single gene testing in the color key of Figure 2; and 2 of the denominators in eTable 3 in Supplement 1 were incorrect (for surgical samples and others in the RNA analysis column). This article has been corrected.
